# Exploring the Effects of Mindfulness on Adolescent Depression—Findings from a Longitudinal Study

**DOI:** 10.3390/healthcare13080906

**Published:** 2025-04-15

**Authors:** Filipa Ćavar, Josipa Mihić, Goran Milas

**Affiliations:** 1 Institute of Social Sciences “Ivo Pilar”, 10000 Zagreb, Croatia; filipa.cavar@pilar.hr; 2 Faculty of Education and Rehabilitation Sciences, University of Zagreb, 10000 Zagreb, Croatia; josipa.mihic@erf.unizg.hr

**Keywords:** mindfulness, depression, adolescents, mental health

## Abstract

**Background/Objectives:** Adolescence is a critical period for mental health, with depression increasing rapidly and often leading to lifelong consequences. In recent decades, the prevalence of elevated depressive symptoms among adolescents has steadily risen, making it a significant public health concern. While research supports the benefits of mindfulness-based practices in reducing adolescent depressive symptoms, the role of trait mindfulness remains underexplored. Although some studies suggest a link between trait mindfulness and lower depressive symptomatology, a longitudinal perspective could provide deeper insights into this relationship. Given adolescents’ heightened vulnerability to mental health issues, understanding the potential causal link between trait mindfulness and depression is crucial for both prevention and intervention efforts. **Methods:** This study examines the relationship between mindfulness and depression in a three-wave longitudinal study of 1618 secondary school students (Males: N = 671, M = 16.4 years, SD = 0.60; Females: N = 947, M = 16.3 years, SD = 0.65) using a random intercept cross-lagged panel model. **Results:** Findings indicate that mindfulness and depression share a substantial proportion of variance (r = 0.48) at the stable trait level, suggesting that sustained attentional focus, a hallmark of mindfulness, is consistently associated with fewer depressive symptoms. At the within-person level, momentary deviations from stable mindfulness levels in the first and second waves were linked to lower depressive symptoms in subsequent waves (β = −0.21, *p* = 0.016; β = −0.44, *p* = 0.03, respectively). These findings suggest that even temporary increases in mindfulness may provide additional protection against depression. **Conclusions:** Overall, the results suggest that trait mindfulness is associated with a reduced risk of developing depressive symptoms at both the between-person and within-person levels. Specifically, adolescents with higher stable levels of mindfulness tend to report fewer depressive symptoms over time, and even momentary increases in mindfulness beyond an individual’s typical level are linked to reductions in subsequent depressive symptoms. These findings highlight the potential of mindfulness-based interventions in mitigating adolescent depression and underscore the importance of cultivating mindfulness as a protective factor during this critical stage of development.

## 1. Introduction

### 1.1. Prevalence and Impact of Adolescent Depression

Adolescent mental health problems have become a growing public health concern, with research emphasizing the heightened vulnerability to stress during this period as a key contributor to mental health disorders [1,2]. The prevalence of depression rises sharply in adolescence, reaching nearly 20% by age 18 [3]. Its onset during this critical stage has lasting consequences, leading to long-term mental health impairments [4]. However, focusing solely on clinical diagnoses provides an incomplete understanding of the issue, as it excludes individuals who experience significant depressive symptoms but do not meet the criteria for depressive disorder. These adolescents face a substantially higher risk of developing clinical depression in early adulthood [5,6] and are more susceptible to adverse outcomes such as substance misuse, risky or criminal behavior, and poor physical health later in life [7]. Alarmingly, the prevalence of elevated depressive symptoms—an indicator of increased risk for clinical depression—has risen from 24% in the early 2000s to 37% in the following decade [8]. Meanwhile, the prevalence of clinical depression among adolescents has doubled, reaching approximately 25% [9].

Since the pandemic, the prevalence of mental health problems has been rising at an accelerated rate, now affecting approximately 15% of adolescents aged 10 to 19 [10], with problems mainly manifesting in heightened anxiety and depressive symptomatology at the global level of functioning [11]. Notable sex differences have been found considering adolescent depression. The risk of adolescent girls developing major depressive disorder is suspected to be up to three times higher than for their male counterparts, with the risk increasing when considering severe major depressive disorder [12]. This disparity is thought to stem from factors such as socialization patterns [13], the complex interplay of affective, biological, and cognitive vulnerability in interaction with negative life events [14,15], and potentially less effective coping strategies and heightened sensitivity to stress [16], making adolescent girls more vulnerable to depressive symptoms. Regardless of possible underlying biological disparities, early onset of depressive symptoms during adolescence significantly impacts overall functioning and mental health in both sexes [17]. Adolescent depression has been linked to cognitive decline, especially poor executive functioning, leading to difficulties in concentration and decision-making [18], thus indirectly affecting adolescent academic achievement. Additionally, depressive symptoms manifested during this critical developmental period also disrupt adolescents’ social development [4]. Tendency to withdraw from peer activities fosters isolation and strained social interactions, impairing socio-emotional skills [19] and negatively influencing future socio-educational outcomes.

The long-term consequences of adolescent depression are profound. Not only does the onset of mental health problems during this vulnerable period directly affect adolescent mental health and well-being, but it also significantly reduces their resilience capacity and increases the risk of persistent mental health issues later in life [4]. Therefore, the consequences of adolescent depression extend into adulthood, making the person susceptible to a higher risk of recurrent depressive episodes, developing comorbid internalized problems [4], and substance misuse behaviors [20]. These findings underscore the urgent need for early identification and intervention to mitigate long-term adverse outcomes of adolescent depression.

Despite the high prevalence and substantial proportion of adolescents experiencing depressive symptoms at a subclinical level [21,22], many do not receive adequate psychoeducational resources or mental health support. Considering the heightened stress sensitivity of adolescence, it is crucial to examine the risks and protective factors influencing adolescent mental health and to understand the underlying mechanisms and dynamics. Such insights are essential for promoting effective mental health intervention strategies, especially within the educational setting.

### 1.2. Mindfulness and Adolescent Mental Health

Mindfulness, characterized as maintaining a present-moment awareness without judgment, has gained recognition as a promising factor in promoting mental well-being [21]. Brown and Ryan [22] define mindfulness as an inherent tendency to be attentive and aware of present-moment experiences, highlighting its role as a stable psychological trait that varies between individuals. In contrast, state mindfulness refers to momentary awareness that can fluctuate or be intentionally cultivated. Bishop et al. [23] extend this understanding by proposing a two-component model encompassing both attentional regulation and an open, accepting orientation toward experience. This conceptual distinction informs the design of mindfulness-based interventions (MBIs), suggesting that mindfulness can be both cultivated over time (as a trait) and applied flexibly in daily life (as a coping resource). Recognizing these dimensions provides a more comprehensive approach to developing intervention strategies that simultaneously foster mindfulness and resilience capacities and situational coping strategies relevant for managing emotional distress, which is still insufficiently understood.

Research suggests that mindfulness reduces depression in adolescents through interrelated psychological, neurological, and behavioral mechanisms [24]. These mechanisms enhance emotional regulation, reduce rumination, and promote resilience, ultimately mitigating depressive symptoms. Mindfulness strengthens emotional regulation by increasing awareness of emotions without immediately reacting to them. By increasing awareness of emotions without immediate reactivity, it fosters self-regulation, emotional resilience, and cognitive flexibility—processes essential for managing depressive symptoms [22]. Additionally, mindfulness helps adolescents shift from automatic, repetitive negative thinking patterns to a present-focused, nonjudgmental perspective, reducing cognitive distortions that exacerbate depression [25,26].

Higher levels of trait mindfulness have been associated with lower levels of depressive affect among adolescents. Moreover, mindfulness is shown to enhance self-compassion, cognitive flexibility, and emotion regulation [27], which are crucial factors in maintaining psychological well-being and fostering mental health among high school adolescents [28]. Its positive effects extend to reducing perceived stress [29] and psychosomatic symptoms in adolescents [30].

Given the benefits mindfulness has in overall mental health promotion, incorporating mindfulness-based programs as part of the core educational curriculum has gained attention as a preventive strategy for various mental health issues. Mindfulness-based interventions (MBIs), have been implemented in school settings to address mental health concerns among adolescents, showing the promising effects of MBIs in improving the mental health and well-being of youth, which only highlights their potential in alleviating adolescents’ depressive symptoms [31,32]. The accessibility and scalability of such programs make them a promising approach to enhancing adolescent mental health on a broader scale, which only further supports the integration of such preventive actions within educational settings [33].

### 1.3. Key Mechanisms and the Effects of Mindfulness

While the interplay of mindfulness mechanisms is not fully understood, their benefits are more certainly undisputed. At the cognitive level, mindfulness is assumed to de-automatize negative automatic thinking patterns [26] frequently seen in depressive symptomatology. Purposefully shifting the attention to inner experiences, while simultaneously maintaining a non-judgmental attitude to the same, is suggested to reduce rumination and enhance emotional awareness. Thus, recognizing and disengaging the maladaptive cognitive and, consequently, behavioral patterns [25,26] are proposed to be the core changes of mindfulness training concerning depressive symptomatology. Notably, its effect on rumination is of especial importance when considering effective interventions for reducing adolescent depression [34].

Underlying mindfulness mechanisms certainly are intertwined and, following the ABC model [35], simultaneously affect psychological, neural, and behavioral outcomes. Non-reactivity along with non-judgement, are believed to be important mindfulness mechanisms for managing dysphoria and low mood [36]. Non-reactivity to inner experiences is identified as a key mindfulness facet that protects against rumination and negative bias [36]. Moreover, neural imaging has linked this mechanism to reduced insula activation when inhibiting negative stimuli, thus suggesting non-reactivity to inner experiences not only de-automatizes negative thinking but also reduces automatic emotional responding [36]. By strengthening all aspects of emotional intelligence—recognition, expression, and control—mindfulness plays a significant role in reducing adolescent depression [37].

### 1.4. Longitudinal Perspectives on Trait Mindfulness and Depression

Longitudinal studies provide a more comprehensive insight into the mindfulness–depression relationship during adolescence. Not only does mindfulness negatively predict depressive symptoms over time [38], higher levels of trait mindfulness are also associated with lower levels of depressive symptoms that are usually elevated during academically stressful periods [39]. The long-term benefits of mindfulness are seen across various life domains. Along with the beneficial effects that mindfulness has on mental health while experiencing academically challenging periods, its protective role is especially prominent when experiencing parent/family-related stress [40]. Therefore, long-term mindfulness effects are believed to further adolescents’ emotional resilience [41], thus promoting their mental well-being.

Understanding the complex relationship between trait mindfulness, which research suggests is still developing during early adolescence [42], and mental health problems across time is essential for developing effective and timely interventions for mental health promotion during adolescence.

### 1.5. The Current Study

Despite growing evidence supporting mindfulness as a protective factor for adolescent mental health, the exact nature and directionality of the relationship between trait mindfulness and depression remain insufficiently understood. While research indicates that mindfulness is associated with lower depressive symptoms, most studies rely on cross-sectional or short-term experimental designs, which fail to capture within-person fluctuations over time [27,43]. Furthermore, although mindfulness-based interventions have been shown to alleviate depression [44], it remains unclear whether depression itself contributes to lower mindfulness levels, suggesting the possibility of a bidirectional causal relationship.

A key theoretical question concerns the distinction between trait mindfulness and mindfulness practice. Since trait mindfulness represents a relatively stable disposition rather than the direct effects of intervention or the immediate mindful state, it is unclear whether its association with depression stems from enduring personality traits or momentary fluctuations that share a common cause or influence one another. One possibility is that cognitive focus enhances awareness and reduces the negative and mostly automatic thought patterns characteristic of depression. Conversely, depressive affect may impair cognitive awareness, thus increasing the ordinarily automatic thinking and diminishing mindfulness. Finally, recognizing the interconnected mindfulness dimensions while considering its role on depressive symptoms provides needed comprehensive understanding of underlying mechanisms, later important in developing preventive interventions.

To address this theoretical gap, the present study employs a random intercept cross-lagged panel model (RI-CLPM) to examine the reciprocal relationship between mindfulness and depressive symptoms among adolescents over time. This approach distinguishes stable, trait-like differences from within-person fluctuations, offering a more nuanced understanding of their dynamic interplay [45]. Specifically, this study aims to address the following research questions:

1. To what extent is the association between mindfulness and depression driven by stable traits versus momentary deviations? In other words, how much of the shared variance is attributable to individual differences versus within-person fluctuation?
2. How strong are the cross-lagged effects suggesting potential causality, and in which direction do they primarily point—from mindfulness to depression or vice versa? Additionally, is there evidence of reciprocal causation, where mindfulness and depression mutually influence each other over time?

## 2. Method

### Participants & Procedure

A three-wave longitudinal design was employed to investigate the dynamic relationship between mindfulness and depression. Data were collected via a mobile application from a sample of students attending 17 secondary schools in Zagreb, Croatia, as part of the *Longitudinal Adolescent Stress Study* project. The study spanned from spring 2022 to spring 2023. Croatia has three types of secondary schools: gymnasiums, vocational schools, and art schools. Gymnasiums provide a broad general education and prepare students for higher education. Vocational schools focus on equipping students with specialized skills and competencies for specific occupations, often leading directly to employment after graduation. Art schools nurture students’ artistic talents and creativity. Schools were selected using proportional random sampling based on student enrollment numbers. Stratification was applied according to the school’s dominant orientation, resulting in the initial selection of 18 vocational schools and 10 gymnasiums out of 51 public schools. Art schools were excluded, as they enroll only about 3% of students, who can also attend another secondary school in parallel. Approval for the selected schools was obtained from the relevant ministry. Schools were then contacted, and 15 agreed to participate in the first wave, with two additional schools joining in the second, bringing the final sample to 10 vocational schools and 7 gymnasiums. In each school, data collection included five first-year and five second-year classes. While some schools were unable to participate due to tight academic schedules, this did not significantly impact the sample structure. Gender representation remained balanced, and vocational school students comprised approximately 60% of the sample, closely reflecting their proportion in the overall population. Analyses were conducted on a subsample of students who met the criteria for careful responding and provided valid responses in at least two of the three waves, yielding a total of 1618 participants. Since gymnasium students—where females are overrepresented—tended to respond more carefully, the sample shifted slightly toward a higher representation of both females and gymnasium students. The initial data collection took place in March 2022, followed by two additional assessments at approximately six-month intervals. The first wave included 1308 participants, the second wave 1186, and the third wave 1346 (Table 1).

At the start of the study, participants were in either the first or second grade of secondary school, with ages ranging from 14 to 19 years (M = 16.32 years, SD = 0.63). Of the total sample, 671 students (41.5%) were male (mean age at baseline = 16.4 years, SD = 0.60), and 947 (58.5%) were female (mean age at baseline = 16.3 years, SD = 0.65). Vocational school students constituted the majority in all waves, with proportions closely matching those in the general population: 62.2% in the first wave, 62.7% in the second, and 53.7% in the third.

The number of students varied across waves, primarily due to the availability of schools for testing. Some schools were unable to participate in certain waves because of their busy academic schedules. Student non-participation was mainly due to illness-related absences on the day of testing. Fewer than 5% of students who were present at school refused to participate. There was minimal classic attrition or dropout from the study. Many students who were absent in the first wave due to illness participated in the second and third waves, and those absent in the second wave were likely to return in the third. Absences were mostly due to incidental factors, such as illness, rather than any systematic cause. Therefore, the likelihood of sample or measurement bias resulting from selective attrition was minimal, as supported by the missing values analysis.

Before the assessment, participants were briefly informed about the project’s objectives and research focus, after which they provided informed consent. For those under the age of 15, parental or guardian consent was also obtained via email, in accordance with the relevant ethics code and regulations. Additionally, the research had been prior approved by the Ethics Committee and found to be in the accordance with relevant laws and ethics code regulations.

## 3. Measures

### 3.1. Mindfulness

The Mindful Attention Awareness Scale for Adolescents [46,47] was used as a measure of trait mindfulness. Participants rated their everyday experiences on items such as “*I find it difficult to stay focused on what’s happening in the present*” on a scale from 1 (almost always) to 6 (almost never). Scores were calculated as an average of all 14 items. This mindfulness measure showed high internal consistency across all assessment points (α = 0.90 to 0.91) on the sample of Croatian adolescents.

### 3.2. Depression

Depressive symptom severity was assessed using the PHQ-9 [48], a brief self-report measure based on the nine DSM-IV diagnostic criteria. The PHQ-9 primarily measures depressive symptoms rather than clinical depression as a formal diagnosis. It is a self-report tool that assesses the frequency of nine specific depressive symptoms over the past two weeks, based on the criteria outlined in the *Diagnostic and Statistical Manual of Mental Disorders* (DSM-IV) for major depressive disorders. While the PHQ-9 can be used as a screening tool to identify potential depression and help assess the severity of depressive symptoms, it is not a diagnostic tool by itself. Participants rated items such as “*Little interest or pleasure in doing things*” on a scale from 0 (not at all) to 3 (nearly every day). Scores were calculated as the sum of all nine items. The measure demonstrated high internal consistency across all three assessment points (α = 0.88 to 0.90).

### 3.3. Analytic Strategy

In each wave, some data were missing due to factors such as careless responding, premature termination, or student absence on the day of testing. To assess whether the missing data were completely at random (MCAR), we conducted an expectation–maximization (EM) analysis using SPSS 27 (IBM, Armonk, NY, USA, 2020). Little’s MCAR test [49] yielded a chi-squared value of 68,765 (df = 39, *p* = 0.002), leading to the rejection of the MCAR hypothesis. Since the missing at random (MAR) assumption cannot be directly tested—because it involves unobserved data [50]—we used indirect evidence to rule out the possibility that data were missing not at random (MNAR). Specifically, we applied a pattern mean difference approach [51] and performed binomial regression analyses to examine whether missingness in any wave was associated with depression or mindfulness scores from other waves. After applying Bonferroni correction, none of the 12 analyses yielded a statistically significant odds ratio (*p* ranged from 0.013 to 0.088). To further evaluate the MAR assumption, we compared the EM imputed data with the observed data distributions for all six variables using Kolmogorov–Smirnov tests and t-tests. After applying Bonferroni correction to control for group-level error, no significant differences were found in either the distribution shape or central tendency. These results support the plausibility of the MAR assumption. Both analyses suggest that missingness in any given wave was not systematically related to depression or mindfulness scores in other waves, providing strong evidence that the data were likely missing at random.

Before proceeding with further analyses, we sequentially tested three types of longitudinal measurement invariance using the multiple-group CFA approach [52]: configural, metric (weak factorial), and scalar (strong factorial). Following recommendations by Putnick and Bornstein [53], model fit was evaluated using two indices: the root mean square error of approximation (RMSEA) and the comparative fit index (CFI). Measurement invariance was considered supported if the change in CFI between nested models was less than 0.01 [54] and the change in RMSEA was less than 0.015 [55]. Both instruments were treated as unidimensional scales composed of ordinal items. CFAs were conducted using JASP [56] based on the R package lavaan [57].

To test the hypothesis of reciprocal causation between mindfulness and depression, we employed the random intercept cross-lagged panel model (RI-CLPM) [45]. Since both depression and trait mindfulness have stable dispositional components, RI-CLPM offers greater precision than the traditional cross-lagged panel model (CLPM) by distinguishing within-person from between-person variance, thereby reducing potential bias [58]. Model fit was evaluated using several criteria: the χ^2^ goodness-of-fit statistic, which measures the discrepancy between the obtained and fitted covariance matrices, as well as the Tucker–Lewis index (TLI), CFI, and RMSEA. Given the sample size, model fit was considered good if TLI and CFI values were close to 0.95 and RMSEA was below 0.07 [59,60]. All analyses were conducted using SPSS 27 [61] and Amos 27 [62].

## 4. Results

Before conducting the analyses, we evaluated the longitudinal measurement invariance of both instruments across waves. As shown in the Supplementary Tables (Tables S1 and S2), both the MAAS-A and PHQ-9 satisfied the criteria for metric and scalar invariance. Descriptive indicators for depression and mindfulness across measurement points are displayed in Table 2.

The mean depression scores based on the PHQ-9 in the Croatian sample were significantly higher than those reported in comparable adolescent samples from Spain [63] (t = 5.34, *p* < 0.001) and Norway [64] (t = 12.81, *p* < 0.001). This finding suggests either a greater susceptibility to depression among Croatian adolescents or cultural differences in how the questions are interpreted. Cross-cultural research on emotion regulation suggests that cultural norms strongly influence how emotional distress is experienced and reported [65,66], where cultures with more restrictive norms around emotional expressivity may show greater internalization of distress and higher self-reported depressive symptoms.

Similarly, mean mindfulness scores in the sample of Croatian adolescents based on MAAS-A were somewhat lower than of those in a comparable Dutch adolescent sample [46] (t = 18.04 *p* < 0.001) in all measurement points, suggesting overall lower levels of trait mindfulness among Croatian adolescents. These differences may reflect cultural variation in emotion regulation strategies and exposure to mindfulness promotion. For example, mindfulness practices may be more culturally introduced in countries like the Netherlands, where socio-emotional learning is more integrated into educational settings, thereby enhancing trait mindfulness among adolescents.

Reliability, as measured by the alpha coefficient, was consistently high for both instruments across all three waves, with values around 0.90.

Correlations between measures of the same constructs across different waves were relatively strong (Table 3). Test–retest reliabilities ranged from 0.62 to 0.69 for depression and 0.55 to 0.76 for mindfulness. The association between reported depression and mindfulness was moderately strong and negative, with correlations between −0.45 and −0.68.

**Figure 1:**
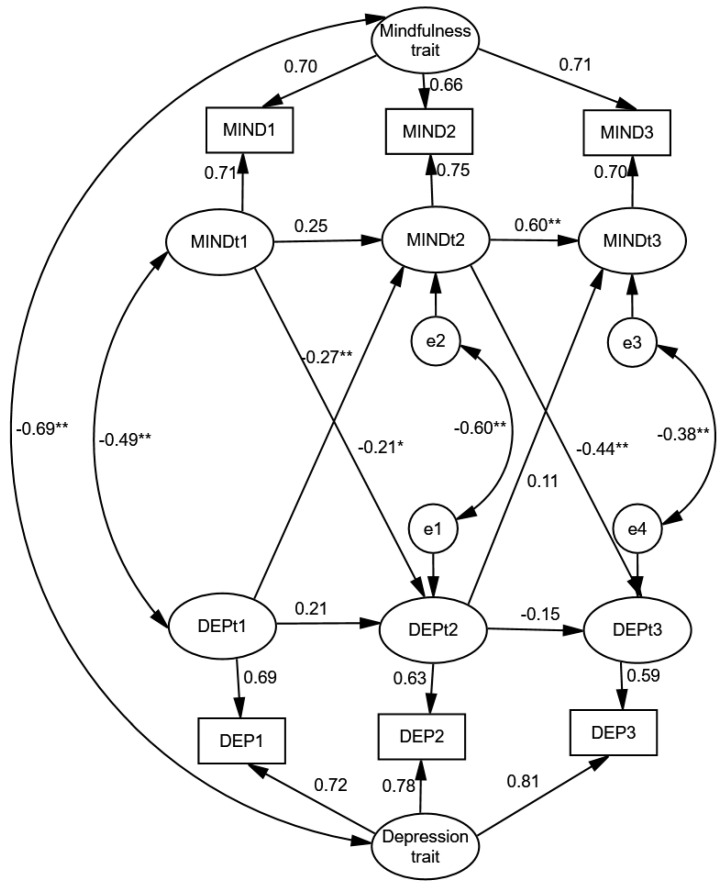
Standardized random intercept cross-lagged panel model solution describing the longitudinal relationship between depression and mindfulness in adolescents (N = 1618). Note: Model fit: χ^2^(1) = 4.087, *p* = 0.043; RMSEA = 0.044 (90% CI 0.006–0.091); CFI = 0.999; TLI = 0.974. The figure displays standardized coefficients. ** *p* < 0.01; * *p* < 0.05.

Figure 1 indicates that the inverse association between mindfulness and depression is predominantly driven by a strong correlation between their stable underlying traits (r = −0.69; *p* < 0.001). In contrast, within-person correlations were notably weaker. Specifically, individual deviations from stable trait levels exhibited moderate negative concurrent correlations across all three waves (ranging from −0.38 to −0.60), implying potential short-term causal effects or coupling influenced by a common causal factor.

Furthermore, cross-lagged coefficients—assessing the possible causal impact of deviations in one construct on another—were statistically significant for mindfulness. Deviations in mindfulness predicted subsequent deviations from the average level of depressive symptoms (β_m1d2 = −0.21, *p* = 0.016; β_m2d3 = −0.44, *p* = 0.003). Additionally, a significant cross-lagged coefficient for depression predicting mindfulness was found (β_d1m2 = −0.27, *p* = 0.002), implying a possible reciprocal relationship in which these opposing constructs mutually diminish each other.

The autoregressive coefficients, which reflect slowly changing factors or autoregressive traits [67], were both non-significant for depression. This finding suggests that deviations from an individual’s typical level of depression at one time point do not predict deviations at the next. In other words, short-term within-person fluctuations in depression appear to lack temporal stability. Two possible explanations are that these variations are primarily driven by external factors, or that the chosen time interval between assessments may be either too short or too long to capture stable patterns. In contrast, deviations from mindfulness traits in the second wave were predictive of similar deviations in the subsequent wave (0.60; *p* < 0.001). Overall, while the results indicate that mindfulness and depression share a substantial proportion of common variance at the level of stable traits, there is some evidence that mindfulness may reduce depression at the within-person level. The evidence for depression’s impact on mindfulness and the presence of reciprocal effects remains weak and inconsistent.

## 5. Discussion

Previous research has established that mindfulness practice can reduce depressive symptoms, and that trait mindfulness is consistently linked to lower depression levels. However, some theoretically relevant questions remained unaddressed. This study aimed to address the nature of the relationship between trait mindfulness and severity of depressive symptoms in adolescents, distinguishing the stable trait-level associations versus momentary deviations, and exploring possible (reciprocal) causality between constructs. In this longitudinal study using RI-CLPM analysis, we found that trait mindfulness and depression shared nearly half of their variance due to stable dispositions. Additionally, concurrent correlations between momentary deviations from these stable traits accounted for 14% to 36% of the variance, depending on the wave. Within-person effects of momentary mindfulness on subsequent depression were also observed, explaining 4% to 20% of the variance. In contrast, the reciprocal effect—where momentary depression reduces subsequent mindfulness—was inconsistent and considerably weaker.

Studies concerning mindfulness and adolescent mental health outcomes suggest its benefits in predicting and reducing depressive symptoms [38,68]. Examining the stable trait-level association between mindfulness and adolescent depression, our results suggest that adolescents with inherently higher mindfulness levels tend to consistently experience fewer depressive symptoms. These findings align with previous research indicating that trait mindfulness is inversely related to psychological distress and internalizing symptoms in adolescents [68].

At the within-person level, we observed a moderate negative correlation between deviations from stable traits across all three waves. This suggests that fluctuations in adolescents’ mindfulness over time are inversely related to changes in their depressive symptoms, suggesting its beneficial effect. While longitudinal studies on this specific relationship remain limited, the literature generally supports the protective role of mindfulness in mitigating internalizing problems in adolescence [38].

While our findings suggest of an overall protective role of mindfulness, concerning both stable trait-like levels and momentary fluctuations in adolescents’ depressive symptoms, the underlying mechanisms remain uncertain. A possible explanation of such favorable effects of mindfulness on adolescent depression, relies on the fact that mindfulness successfully de-automizes and reduces automatic, habitual, and usually negative cognitive and behavioral responses [69], with rumination being one of main precursors to depressive symptomatology. Mindfulness facets such as acting with awareness, non-judgement, and non-reactivity to inner experiences are presumed to be the key factors in explaining the inverse relationship between mindfulness and depression. By reducing rumination tendencies, thus subsequently decreasing depressive symptoms over time [23], mindfulness seems to provide a more conscious and adaptive response to external stimuli [69]. Thus, it acts as both a protective factor against internalized mental health problems [38] and a promoting factor in strengthening emotional resilience [37].

As expected, the association between trait mindfulness and depression is largely driven by stable individual differences. Adolescents who, on average, report higher levels of depression across time also tend to report lower levels of mindfulness across time. However, the relationship between mindfulness and depression extends beyond trait-level associations. Their momentary deviations from the average are also significantly correlated at the same time point, reflecting within-person concurrent associations. This suggests that fluctuations in mindfulness and depression tend to co-occur in the moment, possibly due to short-term effects of mindfulness or the simultaneous activation of related cognitive mechanisms.

Most notably, the cross-lagged effects provide further insight into this relationship. The results indicate that when adolescents experience higher-than-usual mindfulness at a given time point, they are more likely to report lower-than-usual depression six months later. This finding highlights the potential therapeutic impact of mindfulness, even when considering short-term fluctuations around an individual’s typical levels. Thus, mindfulness poses promising benefits considering both regular daily practices aimed at mental well-being promotion, as well as in form of targeted mindfulness-based intervention aimed for higher-risk youth.

Future research could benefit from alternative longitudinal approaches, such as testing at shorter intervals or using experience sampling methodology to better capture dynamic fluctuations and explore the underlying mechanisms linking mindfulness to mental health outcomes. Cross-lagged effects suggest that within-person increases in mindfulness may help reduce subsequent depression. Although there is some evidence for the reverse effect, it is notably weaker. Taken together, these within-person effects and concurrent correlations may represent the upper threshold of mindfulness-based training effectiveness—potentially equipping adolescents with the skills to integrate mindfulness into their daily lives.

## 6. Limitations

This study was conducted on a large and mostly representative sample of high school adolescents. However, several limitations should be noted. First, as a correlational study, it cannot establish causality—any causal interpretations remain speculative. Although longitudinal, the six-month intervals between waves may have been too long to capture short-term within-person causal effects. Additionally, the study does not account for other potentially relevant variables that could moderate, mediate, or confound the relationship between mindfulness and depression. Future research should consider factors such as personality traits or resilience. Lastly, relying solely on self-reporting to measure depression and mindfulness is a potential limitation, as it may introduce various biases, including social desirability bias, response bias, recall bias, and common method bias. Future research could enhance validity by cross-validating self-reports with alternative assessment methods, such as observer or peer ratings. Finally, additional variables such as life stress or mental well-being are not accounted for in this research, but could provide a broader understanding of found relationships. Thus, additional research incorporating stress measurement and coping strategies is of valuable interest considering the role of mindfulness in the current mental health state.

## 7. Conclusions

Mindfulness has emerged as a promising protective factor in mitigating depressive symptoms among adolescents. However, the exact nature and direction of the relationship between trait mindfulness and depression remain insufficiently understood. A deeper understanding of the longitudinal dynamics of this relationship is essential for identifying key cognitive mechanisms and informing effective prevention strategies.

Our findings suggest a dual role of mindfulness in relation to depression. First, mindfulness and depression are primarily linked at the level of stable dispositions, indicating that adolescents with inherently higher trait mindfulness experience fewer depressive symptoms. Second, momentary deviations from these stable traits further reinforce the negative association, suggesting that fluctuations in mindfulness also contribute to reduced depressive mood over time.

Thus, mindfulness appears to inversely relate to adolescent depression, offering benefits both as a stable trait and through within-person fluctuations over time. This dual nature aligns with theoretical frameworks that distinguish between trait- and state-like dimensions of mindfulness [22,34], and it carries important implications for intervention design. Specifically, prevention strategies may be optimized by integrating both components, thus sustaining trait development—such as structured mindfulness training programs—and state-level coping, by teaching adolescents to apply mindfulness practices in response to momentary stressors. Further research is needed to specify the mechanisms that connect these two levels; however, our findings highlight the importance of incorporating mindfulness dimensions into effective mental health promoting interventions aimed at youth.

## Figures and Tables

**Table 1:** Socio-demographic composition of the sample across waves.

	N	Biological Sex		Age		School Type	
Time Point		% Males	% Females	M	SD	% Vocational	% Gymnasium
Time 1	1308	43.27	56.73	16.32	0.63	62.19	37.81
Time 2	1186	40.98	59.02	16.83	0.62	62.70	37.30
Time 3	1346	40.19	59.81	17.35	0.62	53.71	46.29

**Table 2:** Means and standard deviations of depressive symptoms and mindfulness across measurement points.

	Time	M	SD	Min.	Max.	Reliability (α)
Depression	T1	8.82	6.70	0.00	27.00	0.90
	T2	8.97	6.22	0.00	27.00	0.88
	T3	8.34	5.95	0.00	27.00	0.88
Mindfulness	T1	3.66	0.97	1.00	6.00	0.89
	T2	3.77	1.05	1.00	6.00	0.92
	T3	3.74	0.96	1.00	6.00	0.90

**Table 3:** Zero-order correlations between depression and mindfulness across three time points.

	1	2	3	4	5	6
1. Depression t1	—					
2. Depression t2	0.689 ***	—				
3. Depression t3	0.624 ***	0.670 ***	—			
4. Mindfulness t1	−0.595 ***	−0.536 ***	−0.451 ***	—		
5. Mindfulness t2	−0.553 ***	−0.678 ***	−0.552 ***	0.663 ***	—	
6. Mindfulness t3	−0.466 ***	−0.535 ***	−0.609 ***	0.550 ***	0.761 ***	—

Note: *** *p* < 0.001.

## Data Availability

The data that support the findings of this study are available from the corresponding author upon reasonable request.

## Supplementary Materials

The following supporting information can be downloaded at: https://www.mdpi.com/article/10.3390/healthcare13080906/s1, Table S1: Longitudinal Measurement Invariance Results for the Mindfulness Scale (MAAS-A). Table S2: Longitudinal Measurement Invariance Results for the Depression Scale (PHQ-9). Table S3: Zero-Order Correlations between Depression and Mindfulness Across Three Time Points.
