# Supplementary material for: Exploring the Effects of Mindfulness on Adolescent Depression—Findings from a Longitudinal Study
Source: Healthcare (Basel). 2025 Apr 15;13(8):906. doi: 10.3390/healthcare13080906 (PMC12027095; doi:10.3390/healthcare13080906)
Supplement: Supplementary file 1 [file healthcare-13-00906-s001.zip › healthcare-3576228-supplementary.pdf]

Supplement

Table S1

Longitudinal Measurement Invariance Results for the Mindfulness Scale (MAAS-A)

	Model	χ^2	df	p	CFI	RMSEA	$\Delta\chi^2$	df	p	ΔCFI	ΔRMSEA	Invariance
	Configural	1589.747	231	< .001	0.944	0.102						
First wave	Metric	1087.707	257	< .001	0.966	0.076	-502.04	26	.002	-0.022	-0.026	Yes
	Scalar	1356.869	367	< .001	0.959	0.069	269.162	110	< .001	0.007	-0.007	Yes

Note. df = degrees of freedom; CFI = comparative fit index; RMSEA = root-mean-square error of approximation.

Table S2

Longitudinal Measurement Invariance Results for the Depression Scale (PHQ-9)

	Model	χ^2	df	p	CFI	RMSEA	$\Delta\chi^2$	df	p	ΔCFI	ΔRMSEA	Invariance
	Configural	928.326	81	< .001	0.974	0.091						
First wave	Metric	618.271	97	< .001	0.984	0.065	-310.055	16	.003	-0.010	-0.026	Yes
	Scalar	810.53	131	< .001	0.979	0.064	192.259	34	.030	0.005	-0.001	Yes

Note. df = degrees of freedom; CFI = comparative fit index; RMSEA = root-mean-square error of approximation.

Table S3

Zero-Order Correlations between Depression and Mindfulness Across Three Time Points

	1	2	3	4	5	6
1. Depression t1	—					
2. Depression t2	.689***	—				
(p)	(.000)					
3. Depression t3	.624***	.670***	—			
(p)	(.000)	(.000)				
4. Mindfulness t1	-.595***	-.536***	-.451***	—		
(p)	(.000)	(.000)	(.000)			
5. Mindfulness t2	-.553***	-.678***	-.552***	.663***	—	
(p)	(.000)	(.000)	(.000)	(.000)		
6. Mindfulness t3	-.466***	-.535***	-.609***	.550***	.761***	—
(p)	(.000)	(.000)	(.000)	(.000)	(.000)	
